# Is the diagnostic model based on convolutional neural network superior to pediatric radiologists in the ultrasonic diagnosis of biliary atresia?

**DOI:** 10.3389/fmed.2023.1308338

**Published:** 2024-01-08

**Authors:** Xingxing Duan, Liu Yang, Weihong Zhu, Hongxia Yuan, Xiangfen Xu, Huan Wen, Wengang Liu, Meiyan Chen

**Affiliations:** ^1^Department of Ultrasound, Changsha Hospital for Maternal and Child Health Care, Changsha, China; ^2^Department of Ultrasound, Hunan Children’s Hospital, Changsha, China; ^3^Department of Ultrasound, Chenzhou Children’s Hospital, Chenzhou, China; ^4^Department of Ultrasound, The Third Xiangya Hospital of Central South University, Changsha, China; ^5^Department of Ultrasound, Chaling Hospital for Maternal and Child Health Care, Chaling, China

**Keywords:** biliary atresia, ultrasonography, artificial intelligence, convolutional neural network, diagnosis

## Abstract

**Background:**

Many screening and diagnostic methods are currently available for biliary atresia (BA), but the early and accurate diagnosis of BA remains a challenge with existing methods. This study aimed to use deep learning algorithms to intelligently analyze the ultrasound image data, build a BA ultrasound intelligent diagnostic model based on the convolutional neural network, and realize an intelligent diagnosis of BA.

**Methods:**

A total of 4,887 gallbladder ultrasound images of infants with BA, non-BA hyperbilirubinemia, and healthy infants were collected. Two mask region convolutional neural network (Mask R-CNN) models based on different backbone feature extraction networks were constructed. The diagnostic performance between the two models was compared through good-quality images at the image level and the patient level. The diagnostic performance between the two models was compared through poor-quality images. The diagnostic performance of BA between the model and four pediatric radiologists was compared at the image level and the patient level.

**Results:**

The classification performance of BA in model 2 was slightly higher than that in model 1 in the test set, both at the image level and at the patient level, with a significant difference of *p* = 0.0365 and *p* = 0.0459, respectively. The classification accuracy of model 2 was slightly higher than that of model 1 in poor-quality images (88.3% vs. 86.4%), and the difference was not statistically significant (*p* = 0.560). The diagnostic performance of model 2 was similar to that of the two radiology experts at the image level, and the differences were not statistically significant. The diagnostic performance of model 2 in the test set was higher than that of the two radiology experts at the patient level (all *p* < 0.05).

**Conclusion:**

The performance of model 2 based on Mask R-CNN in the diagnosis of BA reached or even exceeded the level of pediatric radiology experts.

## 1 Introduction

Biliary atresia (BA) is a serious hepatobiliary system disease. It is one of the more common causes of persistent obstructive hyperbilirubinemia in infancy, accounting for approximately 25–30% worldwide (1). The clinical manifestations of BA in the early stage, consisting of infantile hepatitis, are very similar to those of non-BA hyperbilirubinemia children. Therefore, these two manifestations are difficult to distinguish, but the treatment is completely different. BA eventually requires surgery (2). However, most of the children with BA die of liver cirrhosis and liver failure within 2 years of age if effective treatment is not promptly provided (3). Therefore, an early and accurate diagnosis and differential diagnosis of BA in children with hyperbilirubinemia are of utmost importance.

Many screening and diagnostic methods are currently available for BA, but early and accurate diagnosis remains a challenge with existing methods (4). Traditional ultrasonography is the most commonly used non-invasive method to examine BA, has high diagnostic efficiency (5), and has the advantages of being non-invasive, simple, and inexpensive. A number of studies have shown that, in the ultrasound diagnosis of BA, the triangular cord sign exhibits high specificity but relatively low sensitivity. However, gallbladder abnormalities demonstrate high sensitivity and specificity (6, 7). The results of a meta-analysis also showed that gallbladder abnormalities are the most sensitive sign of an ultrasonographic diagnosis of BA, and the specificity is also high (8). However, radiologists often need extensive clinical experience to diagnose BA by ultrasound. In some areas with underdeveloped medical conditions, many children with BA are often missed and misdiagnosed because radiologists’ lack of experience in BA diagnosis.

The advantage of image recognition technology by artificial intelligence (AI) compared with traditional methods is that the computer does not rely on the experience of the operator, captures fine-structured lesions pixel by pixel without artificially setting features, automatically extracts features, and identifies and marks suspicious lesions, thereby overcoming the lack of professional knowledge of the operators and improving the diagnostic accuracy of rare diseases to a large extent (9). Convolutional neural network (CNN) is a type of feed-forward neural network that includes convolutional calculations and possesses a deep structure with inherent advantages in image recognition and processing (10). CNN uses the superposition of a series of convolution layers and pooling layers to effectively extract high-level representations of the original image from its pixels and train high-precision classifiers based on these representations. Its unique weight-sharing property and pooling layer greatly reduce the number of parameters that the CNN model needs to train, thus improving the efficiency of the training.

Therefore, in this study, deep learning algorithms were used to intelligently analyze the ultrasound image data of the gallbladder of children with BA, of those with non-BA hyperbilirubinemia, and of healthy infants, and information that is difficult to observe with human eyes was collected. Furthermore, the data were quantified, a CNN-based BA ultrasound intelligent auxiliary diagnostic model was built, and an intelligent diagnosis of BA was realized.

## 2 Methods

### 2.1 Overall design

Gallbladder ultrasound images of children with BA, of those with non-BA hyperbilirubinemia, and of healthy infants were collected and sorted; an auxiliary diagnostic model was built based on CNN; and the model was tested to compare the diagnostic performance between models 1 and 2. The diagnosis of the model with higher efficiency was compared with that of radiologists with different working years in the diagnosis of BA, and the gap in the diagnostic accuracy of BA between the model and pediatric radiologists was explored. This study was reviewed by the medical ethics committee of our hospital (Nos. EC-20231106-3 and HCHLL-2020-18), and the informed consent was signed by the family members of the children.

### 2.2 Study subjects

A total of 597 children with BA (BA group) and 534 children with non-BA hyperbilirubinemia were diagnosed and treated by five medical institutions in the Hunan Province from February 2016 to May 2022, and 498 little healthy infants were also recruited. Healthy infants and infants with non-BA hyperbilirubinemia, involving a total of 1,032 subjects, were classified into the non-BA group. The diagnostic criteria were the following: BA was confirmed by intraoperative cholangiography and pathology. Children with non-BA hyperbilirubinemia were excluded from the BA group by using intraoperative cholangiography or conservative treatment and observed for 3–6 months until the jaundice subsided. All subjects were ≤ 90 days old. The age of infants in the BA group ranged from 5 to 90 days, with a median age of 50 days, including 239 boys and 358 girls. The age of infants in the non-BA group ranged from 10 to 89 days, with a median age of 55 days, including 572 boys and 460 girls.

### 2.3 Ultrasound images acquisition

Different ultrasonic diagnostic systems and different high-frequency ultrasonic probes were used to obtain the largest long-axis ultrasonic images of the gallbladder of all subjects, with each case containing three images, including 3,096 images of the non-BA group and 1,791 images of the BA group, for a total of 4,887 images. The ultrasonic diagnostic systems (probe) included Mindray Resona 7S (L14-5WU, L14-3WU, and L9-3U), Toshiba Aplio 500 (14 L5, 12 L5), Philips Epiq 7C (L12-3), Siemens Sequoia (18 L6 and 10 L4), and SuperSonic Aixplorer (SL10-2). Ultrasound images were directly exported from the ultrasonic diagnostic systems in JPG, TIF, BMP, or DICOM format. The image inclusion criteria were the following: (1) linear array probe, frequency > 8 MHz, rectangular imaging, scanning depth 4–6 cm and (2) clear image, resolution ≥300 × 300 dpi; (3) no markers or scales in the image. The image exclusion criteria were the following: (1) convex array probe or frequency < 7 MHz; scanning depth > 6 cm or < 4 cm; (2) blurred image; (3) the absence of gallbladder; and (4) unclear disease diagnosis.

### 2.4 Construction of the diagnostic model based on CNN

The computer uses the Ubuntu 16.0 operating system (CPU: i7-8750H, GPU: GTX1060, 6G). The intelligent diagnosis model has three main tasks: the detection of the gallbladder in the ultrasound image, the delineation of the region of the gallbladder pixel by pixel, and the diagnosis of BA in the gallbladder. Therefore, this study introduces the mask region convolutional neural network (Mask R-CNN) (11) as the main model architecture, and simultaneously completes the above-mentioned three tasks. The construction of the model is mainly divided into data preprocessing and labeling, feature extraction, region extraction and alignment, and finally mask segmentation and classification.

#### 2.4.1 Dataset division and preprocessing

**Figure 1:**
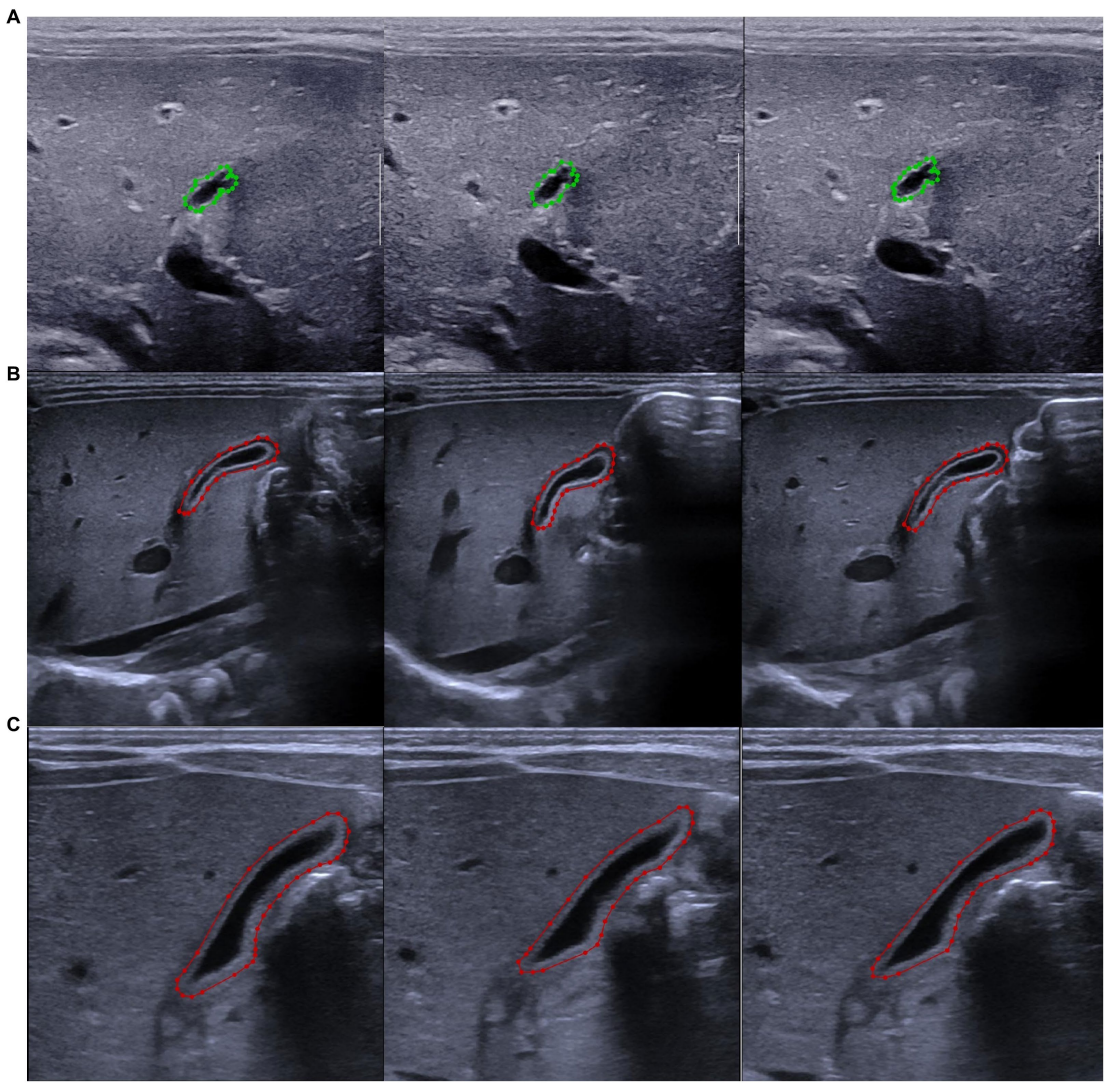
Label of the gallbladder with LabelMe software. **(A)** Marked pictures of the gallbladder in the same patient with biliary atresia. **(B)** Marked pictures of the gallbladder of the same patient with non-biliary atresia hyperbilirubinemia. **(C)** Marked pictures of the gallbladder of the same healthy infant. The biliary atresia group is marked in green, and the non-biliary atresia group is marked in red.

A total of 179 cases were randomly selected from 597 BA children, with each case containing three images, resulting in a total of 537 gallbladder ultrasound images. A total of 196 cases were randomly selected from 1,032 non-BA children, resulting in a total of 588 gallbladder ultrasound images. All selected cases were used as the training set. The remaining cases were used as the test set (a total of 3,762 images). A radiologist with 5 years of experience in pediatric ultrasound used LabelMe image annotation software to manually draw a label that included the entire gallbladder on the training set data, which was checked by another radiologist with more than 15 years of experience in pediatric ultrasound to ensure that the labels were correct (Figure 1). The training set contained 621 Mindray Resona 7S images, 414 Toshiba Aplio 500 images, and 90 Philips Epiq 7C images. The test set contains 2,775 images of Mindray Resona 7S, 327 images of Toshiba Aplio 500, 42 images of Philips Epiq 7C, 582 images of Siemens Sequoia, and 36 images of SuperSonic Aixplorer.

#### 2.4.2 Image feature extraction, region extraction, and alignment

At this stage, two pre-training models were used to extract features from the images. Model 1 used Resnet-101 as the backbone feature extraction network. Model 2 used X-101-32x8d-FPN as the backbone feature extraction network.

ResNet-101 uses skip connections to avoid or weaken network degradation problems caused by gradient disappearance or gradient explosion during deep neural network model training. The feature map of the last layer had strong semantics after the extraction of the gallbladder ultrasound image by ResNet-101, thus finding the difference between the gallbladder and the surrounding tissue, thereby providing effective characteristic information for the detection of the gallbladder and the diagnosis of BA.

X-101-32x8d-FPN inherits the deep convolutional neural network and adopts the “bottom-up” convolution framework to continuously obtain more abstract semantics, as well as a “top-down” process to adapt to the detection of objects of different sizes. The feature maps used for each layer of prediction were integrated with features of different resolutions and different semantic strengths, which completed the detection of the gallbladder with the corresponding resolutions, ensuring that each layer had the appropriate resolution and strong semantic features.

After obtaining the feature map of the ultrasound image of the gallbladder, multiple candidate boundary boxes with different scales for each pixel on the feature map were first constructed based on the regional proposal network. Each bounding box was the candidate region of interest (ROI) containing the gallbladder. The ROI was then mapped back to the original ultrasound image and aligned using the ROI Align module.

#### 2.4.3 Mask segmentation and classification

This stage includes three branches: ROI bounding box coordinate regression, ROI mask segmentation, and classifier. Bounding box coordinate regression completes the calculation of the coordinate offset of the ROI area. It is assumed that the current ROI frame to be processed is described by the vector X,


X=(x,y,w,h)

where x and y are the abscissa and ordinate of the center point of the box, whereas w and h are the width and height of the box, respectively. Its corresponding true value is Y:


Y=(Gx,Gy,Gw,Gh)

If only the two transformations of translation and scaling were considered, the linear transformation Y=WX can be used to model the relationship between the two transformations. As regards this study, the input X was the feature map *Φ* after the fully connected network and the transformation amount t between the current candidate box and the true value box passed in during the training:


$$t=\left(t_{x},t_{y},t_{w},t_{h}\right)$$

The output was the transformation $W^{*}$. Then, the loss function of the transformed network was expressed as follows:


$$L_{\text{Reg}}=\sum_{i}^{N}|t^{i}-W^{*}\Phi \left(X_{i})\right|$$

ROI mask segmentation was designed to obtain the precise location of the gallbladder, and it was realized through a fully convolutional network. A mask with the same resolution as the source image was obtained based on the technology of the convolution mentioned above. The base Mask R-CNN model predicted masks based on a uniform 28 × 28 sized binary grid. It was difficult to accurately represent the effective information of the gallbladder in some cases due to the relatively low resolution of the mask of such size. This project used a discrete cosine transform mask (DCT-Mask) representation to realize the segmentation of the gallbladder (12).

The classifier completed the discrimination of the gallbladder, both positive and negative. In this part, the ROI candidate region extracted above was passed through two fully connected layers for feature fusion, then the classification probability of each category was calculated through the softmax function, and, finally, the classification probabilities were compared with each other to evaluate the category. The corresponding loss of the network was the common cross-entropy loss, recorded as $L_{\text{Cls}}$. When the mask for each ROI was calculated to simplify the training and testing, it was necessary to first determine its category and then calculate the sigmoid cross-entropy loss of a single category based on the information of its category, which was denoted as $L_{\text{Mask}}$. In summary, the loss function used in the network training in this study was expressed as follows:


$$L_{\text{Total}}=L_{\text{Reg}}+L_{\text{Cls}}+L_{\text{Mask}}$$

### 2.5 Model training

The training was based on the fivefold cross-validation method after setting all the parameters to avoid the impact of the division of the dataset on the results, and the gradient descent method was used to minimize the above loss for optimization. First, the training set was randomly divided into five parts: four were used as training data one after another and the remaining one was used as test data for experiments. The corresponding results were obtained for each test, and the average of the five results was used as an estimate of the accuracy of the algorithm. Finally, the trained network was used to extract the gallbladder region of the test images and to distinguish between positive and negative BA.

### 2.6 Model testing

**Figure 2:**
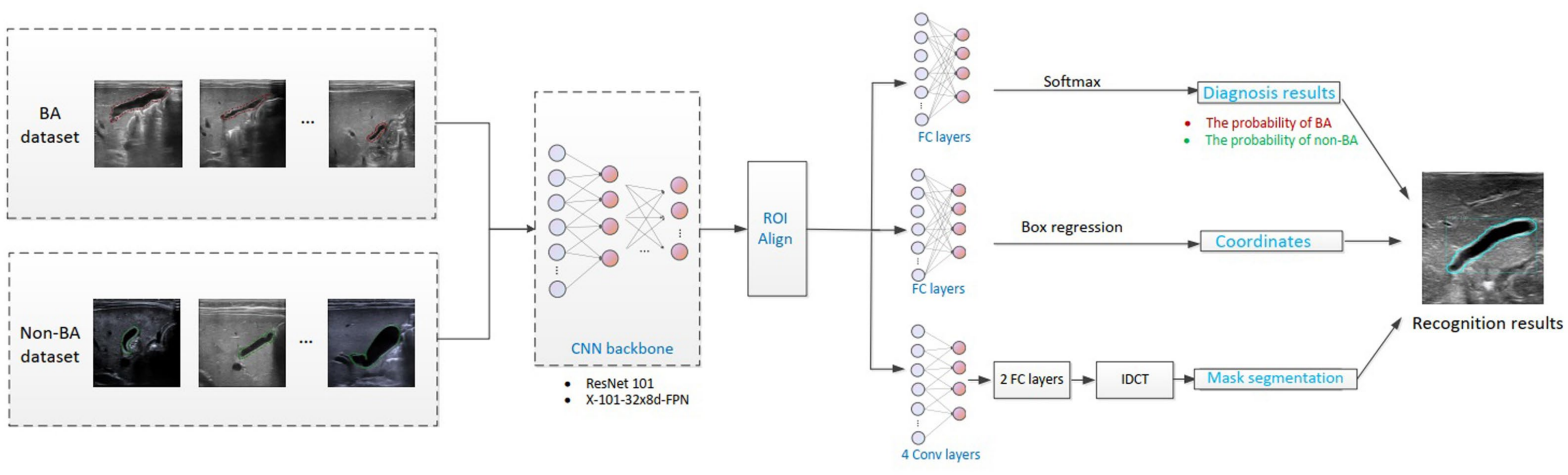
Flowchart of the intelligent analysis of gallbladder images. BA, biliary atresia; CNN, convolutional neural network; IDCT, inverse discrete cosine transform.

**Figure 3:**
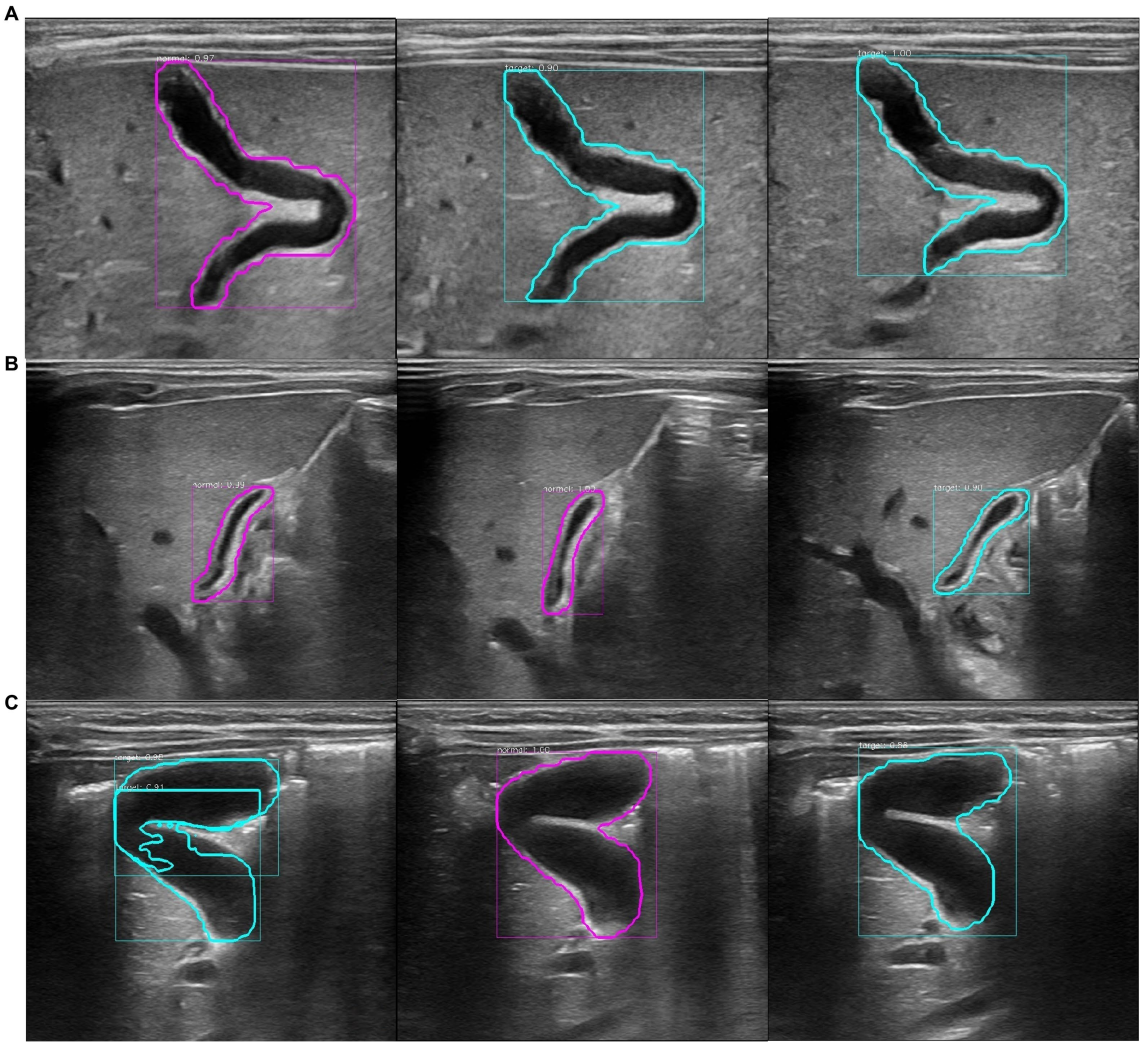
Recognition results of the model at the patient level. The purple circle in the figure represents the recognition result of a single image as non-biliary atresia. The blue–green circle represents the recognition result of a single image as biliary atresia. **(A)** Ultrasound images of the gallbladder of the same patient with biliary atresia. The recognition result shows that the average probability of biliary atresia is approximately 0.64, and the recognition result is correct. **(B)** Ultrasound images of the gallbladder of the same patient with non-biliary atresia and hyperbilirubinemia. The model recognition result shows that the average probability of non-biliary atresia is approximately 0.70, and the recognition result is correct. **(C)** Ultrasound images of the gallbladder of the same healthy infant. The model recognition result shows that the average probability of biliary atresia is approximately 0.62, and the recognition result is incorrect.

Since radiologists usually need to review multiple images of the same patient before providing a diagnosis, in this stage, the test set was used to evaluate the trained model at the image level and the patient level. The flowchart of the gallbladder image analysis is shown in Figure 2. The classification result of the model was considered correct when the model had an image classification probability of >0.5 at the image level. The classification results of the model at the patient level were considered correct when the average classification probability of the three images of the same patient exceeded 0.5 and the classification results were consistent with the actual results; otherwise, the results were considered as incorrect (Figure 3).

Different hospitals have different ultrasound brands, and different styles of ultrasound images have different background noise, which may affect the classification accuracy of the model. Therefore, the classification accuracy of the two models among different brands of ultrasound images was compared at the image level and the patient level.

Most hospitals store their image data in picture archiving and communication systems (PACS) to save data storage space, but the quality of most PACS compressed images (lossy coding) is reduced. Therefore, 103 gallbladder ultrasound images of BA patients and 110 gallbladder ultrasound images of non-BA infants from the PACS of other hospitals were retrospectively collected to evaluate the recognition and classification performance of the two models in the poor-quality images, with one image for each case. These images were compressed by the PACS, and the image size was reduced by approximately 39%. The storage space occupied by the image was reduced by approximately 84%, and the image quality was significantly reduced. These poor-quality images were used to evaluate the diagnostic accuracy of the two models.

### 2.7 Comparison of the diagnostic performance between model and radiologists

The model with higher diagnostic accuracy and more stable performance was chosen to compare with the diagnosis of radiologists. All the pictures in the test set were cut out to remove redundant information, and the images were randomly numbered at the image level and the patient level. Two radiologists (radiologists A and B) with more than 5 years of clinical experience in the diagnosis of pediatric abdominal ultrasound and two radiologists (experts C and D) with more than 15 years of experience in the diagnosis of pediatric abdominal ultrasound were invited. In the absence of the child’s information, the four radiologists provided a diagnosis based on the images of the test set according to the visible morphological information of the gallbladder, including a gallbladder length of ≤15 mm, a gallbladder width of <5 mm, a width ratio of 5.2, unfilled gallbladder, irregular shape, rigid gallbladder wall, mucosa not smooth and intact, and diverticular changes. The interval between two reviews was 2 weeks to avoid the impact of memory on the classification results. The results of the diagnosis were compared with the classification results of the model.

### 2.8 Statistical analysis

Statistical analysis was performed using the SPSS software (version 22.0, IBM Corp., Chicago, IL) and MedCalc Statistical Software version 15.2.2 (MedCalc Software bvba; 2015). ^1^The ratio of the intersection and union (IoU) of the predicted frame and the real frame evaluated whether the extraction of the gallbladder area was successful or not; an IoU of ≥0.5 indicated that the gallbladder area was successfully extracted, and the extraction efficiency was calculated. ROC was used to evaluate the diagnostic performance of the model and the radiologist on BA, and the sensitivity, specificity, positive predictive value (PPV), negative predictive value (NPV), and accuracy were calculated according to the Youden index. The comparison of the diagnostic accuracy of the model between different brands of ultrasound images was performed using the *χ*^2^ test or Fisher’s exact probability test. The comparison of the diagnostic accuracy between the model and radiologists was performed using the *χ*^2^ test or Fisher’s exact probability test. A value of *p* of <0.05 was considered statistically significant.

## 3 Results

### 3.1 Automatic extraction of the model on the gallbladder images

**Figure 4:**
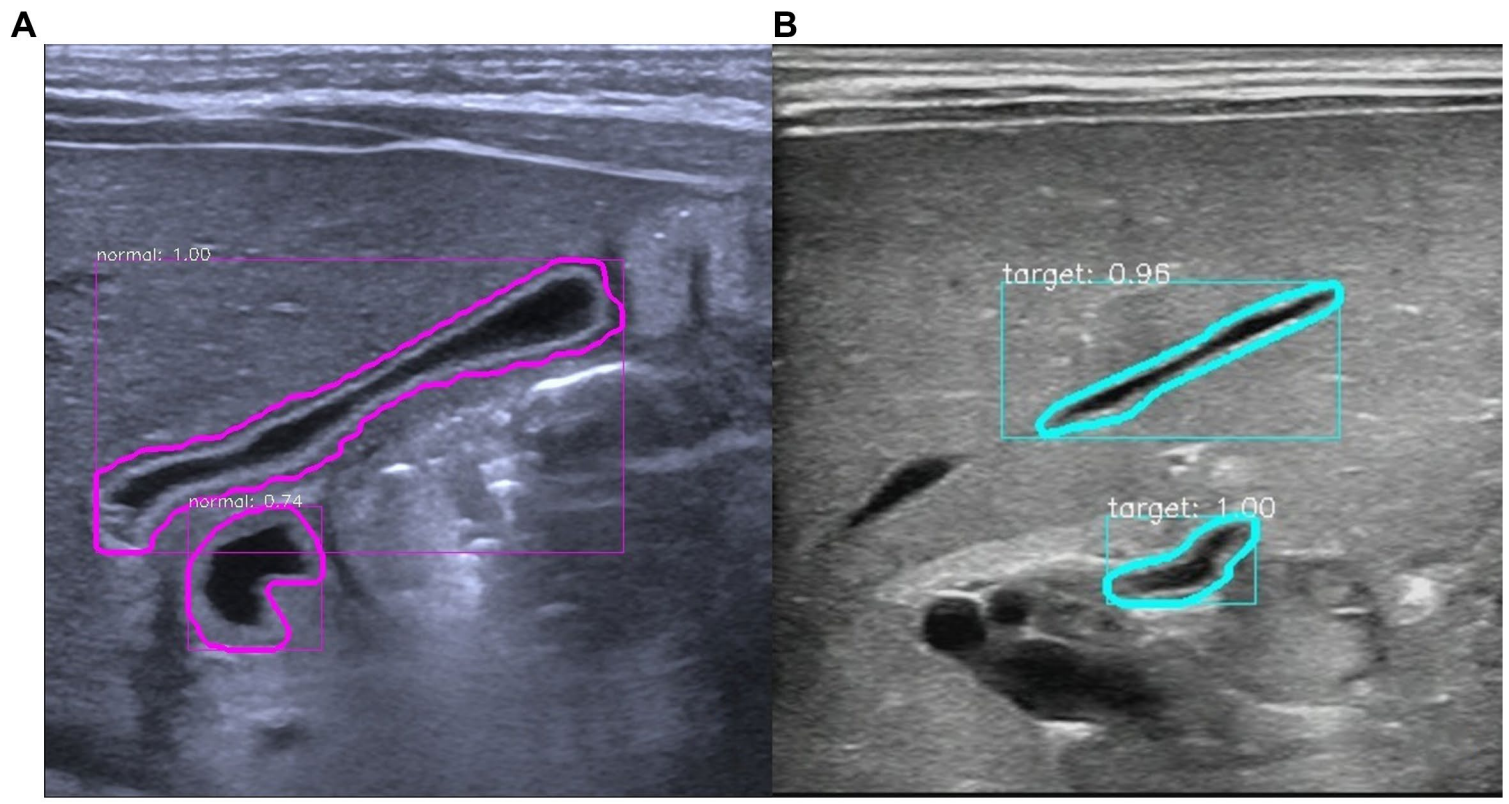
Diagram of the false alarm. **(A)** Gallbladder extraction in a non-biliary atresia infant. The model accurately and automatically extracted the gallbladder organs and revealed a false alarm. The model incorrectly considered the duodenal cavity located below the gallbladder as the gallbladder (lower purple circle). **(B)** Gallbladder extraction in a patient with biliary atresia. The model accurately and automatically extracted the gallbladder organ and revealed a false alarm. The model incorrectly considered the hepatic vein located above the gallbladder as the gallbladder (above the blue–green circle).

Model 1 accurately extracted the gallbladder region in 3,729 images of the test set, and the extraction efficiency was 99.1% (3,729/3,762). However, 155 images showed a false alarm rate of 4.1% (155/3,762). False alarm means that, in the same image, the model accurately detects the real gallbladder, but some non-gallbladder areas were also considered as the gallbladder. Model 2 accurately extracted the gallbladder region from 3,742 images, and the extraction efficiency was 99.5% (3,742/3,762). Nevertheless, 141 images showed false alarms, with a rate of 3.7% (141/3,762). Images with invalid extractions were considered as misinterpreted (Figure 4).

### 3.2 Classification results of BA by different models in the test set

**Figure 5:**
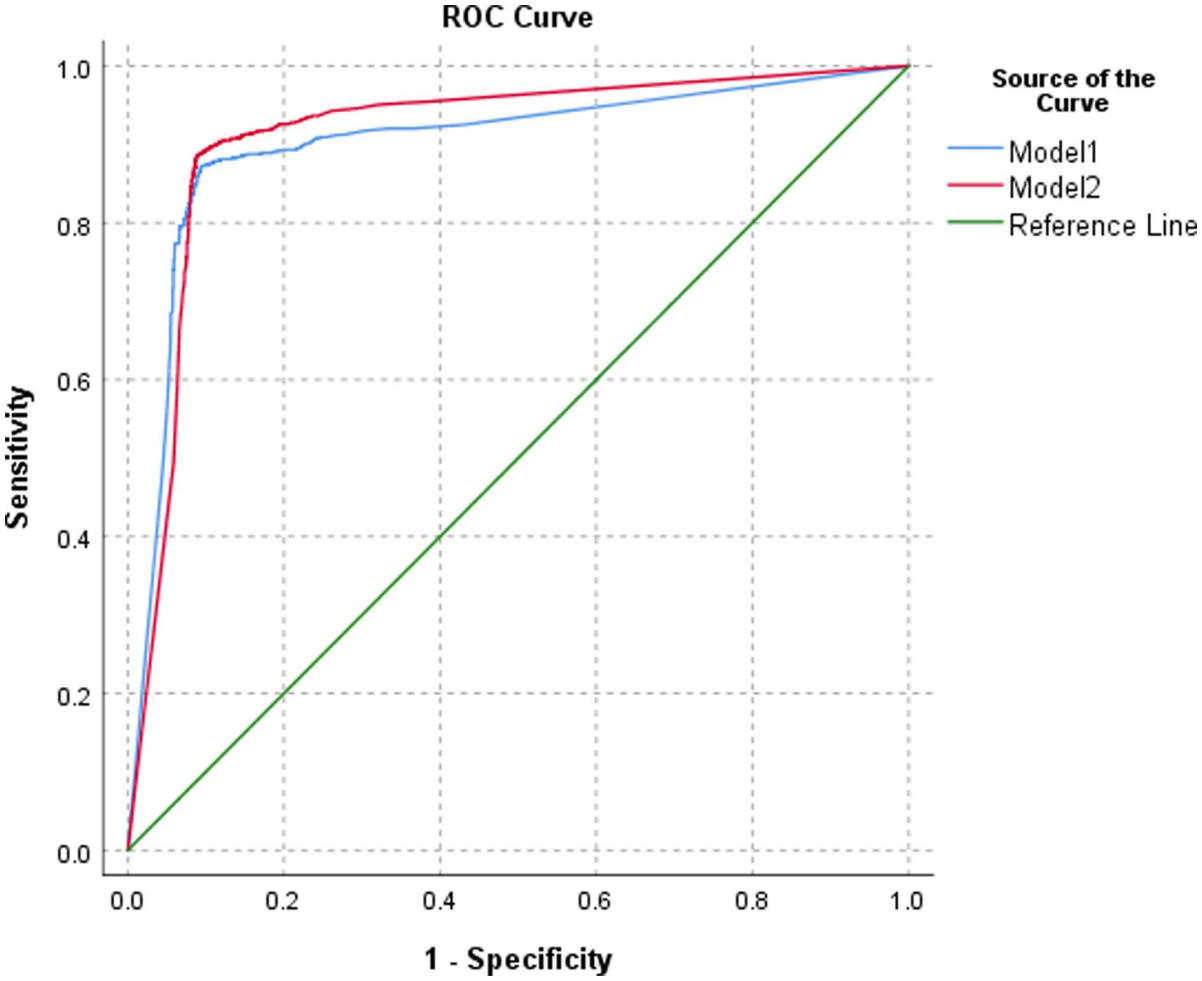
ROC of the performance of different models in classifying biliary atresia at the image level.

**Figure 6:**
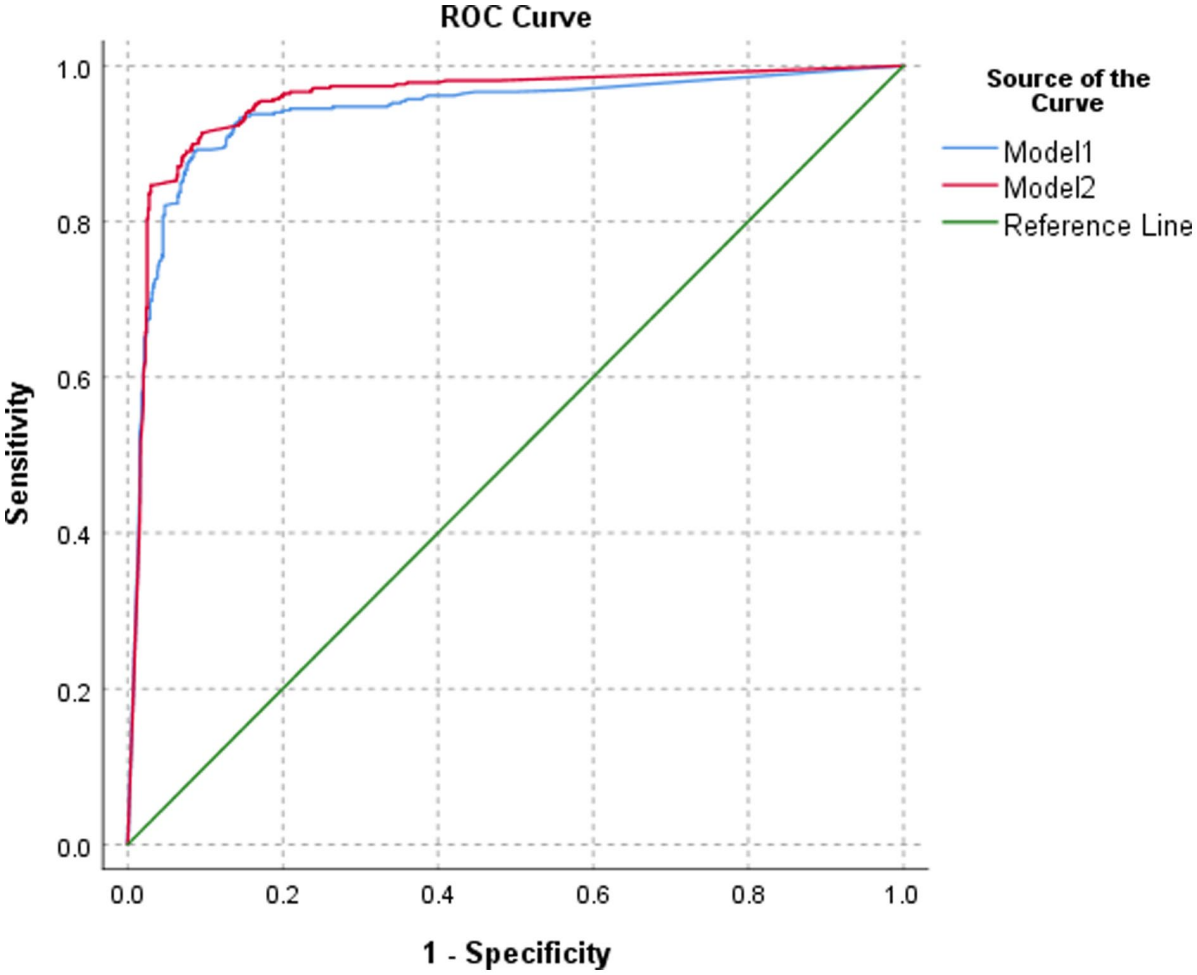
ROC of the performance of different models in classifying biliary atresia at the patient level.

The classification performance of model 2 for BA was slightly higher but statistically significant than that of model 1 in the test set, both at the image level and at the patient level (*p* = 0.0365 and *p* = 0.0459, respectively). At the image level, the AUC of model 2 was 0.913, with a sensitivity, specificity, PPV, NPV, and accuracy of 88.5, 91.2, 83.4, 94.1, and 90.3%, respectively. At the patient level, the AUC of model 2 was 0.956, with a sensitivity, specificity, PPV, NPV, and accuracy of 89.0, 92.5, 85.8, 94.4, and 91.3%, respectively (Table 1; Figures 5, 6).

**Table 1:** Interpretation results of different models for BA in the test set.

Model		AUC	95% CI	*p*-value	Sensitivity	Specificity	PPV	NPV	Accuracy
Image level	AI model 1	0.901	0.889, 0.913	0.0365	87.2%	90.7%	82.4%	93.4%	89.5%
	AI model 2	0.913	0.903, 0.924		88.5%	91.2%	83.4%	94.1%	90.3%
Patient level	AI model 1	0.940	0.924, 0.956	0.0459	88.3%	91.8%	84.3%	94.0%	90.6%
	AI model 2	0.956	0.943, 0.969		89.0%	92.5%	85.8%	94.4%	91.3%

### 3.3 Classification accuracy of the two models on different brands of ultrasound images in the test set

Model 1 had a statistically significant classification accuracy of ultrasound images among different brands at the image level (*P*_1_ = 0.001). Model 2 had similar accuracy in the classification of the ultrasound images of different brands, but the difference in the comparison of these different brands was not statistically significant (*P*_2_ = 0.331). No significant difference at the patient level was found in the classification accuracy among ultrasound images of different brands in either model 1 or model 2 (*P*_1_ = 0.224, *P*_2_ = 0.895) (Table 2).

**Table 2:** The diagnostic accuracy of the two models on different brands of ultrasound images in the test set.

Brand	Image level			Patient level		
	*n*	Model 1	Model 2	*n*	Model 1	Model 2
Mindray Resona 7S	2775	89.6%	90.3%	925	90.7%	91.1%
Siemens Sequoia	582	86.8%	90.0%	194	87.6%	91.2%
Toshiba Aplio 500	327	94.2%	90.8%	109	94.5%	91.7%
Philips Epiq 7C	42	88.1%	90.5%	14	85.7%	100%
SuperSonic Aixplorer	36	100%	100%	12	100%	100%
*p*-value		0.001	0.331		0.224	0.895

### 3.4 Ability of the model in classifying the compressed image

The classification accuracy of model 2 in PACS compressed images was approximately 88.3% and was slightly higher than that of model 1 (86.4%), but the difference was not statistically significant (*χ*^2^ = 0.339, *p* = 0.560). According to the diagnostic performance and robustness of the model, model 2 was chosen for subsequent comparisons.

### 3.5 Performance of model 2 and radiologists in the diagnosis of BA at the image level

**Figure 7:**
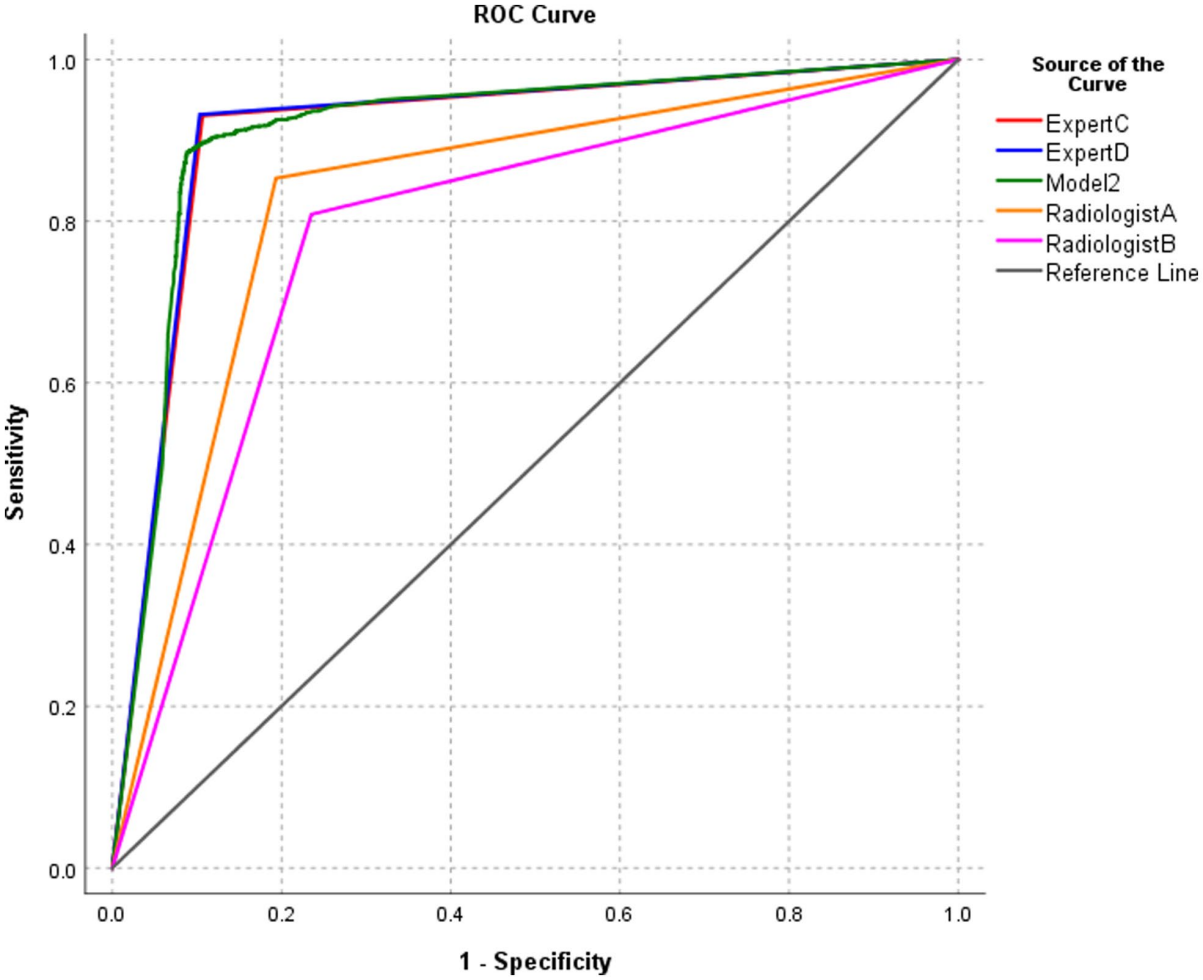
ROC of the performance of model 2 and radiologists in diagnosing biliary atresia at the image level.

The diagnostic performance of model 2 at the image level in the test set was higher than that of the two radiologists with 5 years of clinical experience, and the differences were statistically significant (all *p* < 0.0001). The diagnostic performance of model 2 was similar to that of the two radiology experts with 15 years of clinical experience, and the differences were not statistically significant (Table 3; Figure 7).

**Table 3:** Performance of model 2 and radiologists in diagnosing BA at the image level.

Method	AUC	95% CI	*p*-value	Sensitivity	Specificity	PPV	NPV	Accuracy
AI model 2	0.913	0.903, 0.924		88.5%	91.2%	83.4%	94.1%	90.3%
Radiologist A	0.830	0.816, 0.844	<0.0001	85.4%	80.7%	68.8%	91.7%	82.2%
Radiologist B	0.787	0.771, 0.803	<0.0001	80.9%	76.5%	63.2%	88.9%	78.0%
Expert C	0.912	0.901, 0.923	0.8317	93.1%	89.3%	81.3%	96.3%	90.6%
Expert D	0.914	0.904, 0.925	0.8699	93.2%	89.7%	81.9%	96.4%	90.9%

### 3.6 Performance of model 2 and radiologists in the diagnosis of BA at the patient level

**Figure 8:**
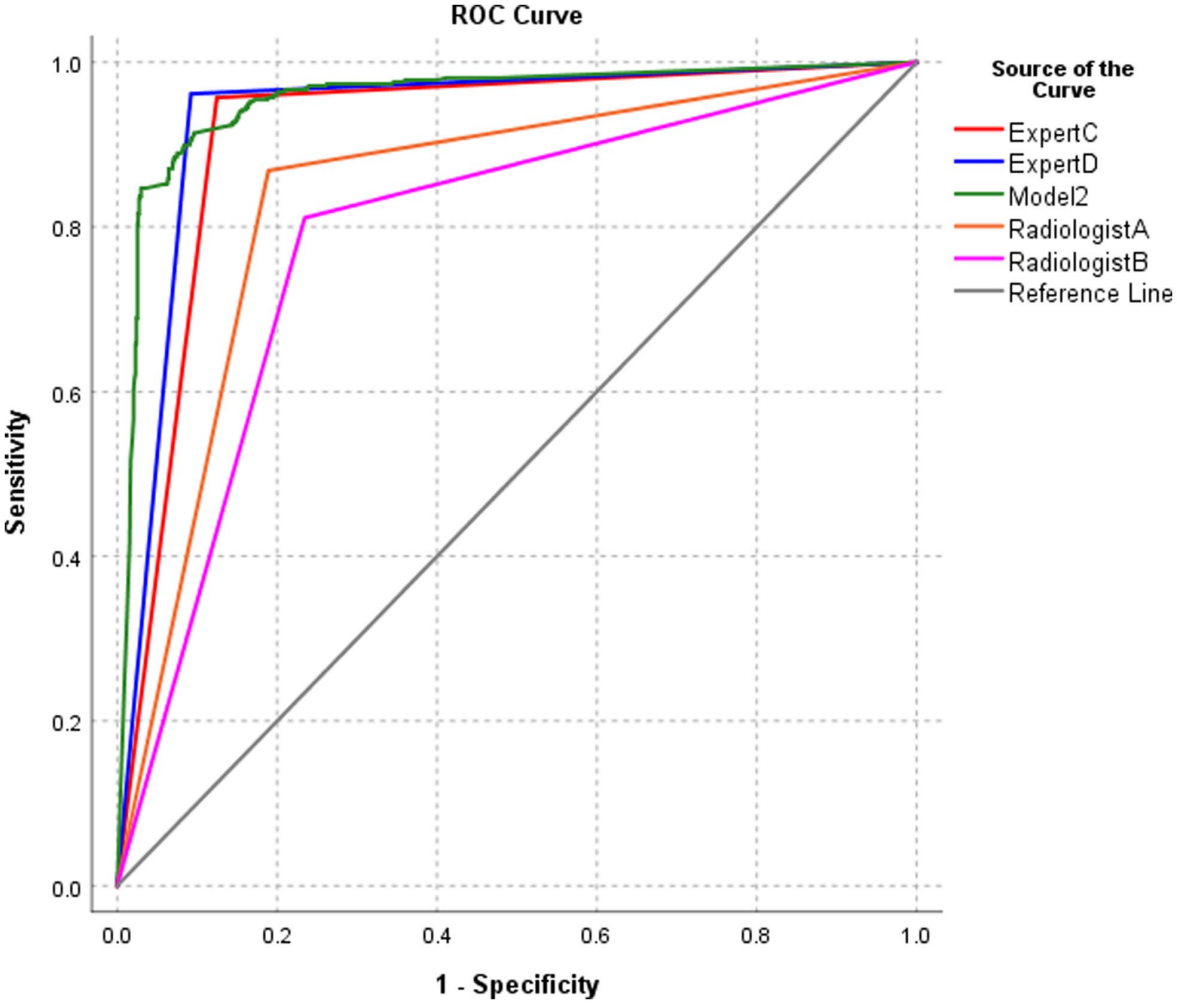
ROC of the performance of model 2 and radiologists in diagnosing biliary atresia at the patient level.

The diagnostic performance of model 2 at the patient level in the test set was higher than that of the four pediatric radiologists, and the differences were statistically significant (all *p-*values were < 0.05) (Table 4; Figure 8).

**Table 4:** Performance of model 2 and radiologists in diagnosing BA at the patient level.

Method	AUC	95% CI	*p*-value	Sensitivity	Specificity	PPV	NPV	Accuracy
AI model 2	0.956	0.943, 0.969		89.0%	92.5%	85.8%	94.4%	91.3%
Radiologist A	0.840	0.815, 0.864	<0.0001	86.8%	81.1%	69.7%	92.5%	83.1%
Radiologist B	0.788	0.761, 0.816	<0.0001	81.1%	79.8%	63.4%	89.4%	80.2%
Expert C	0.916	0.899, 0.934	0.0001	95.7%	87.6%	79.4%	97.6%	90.3%
Expert D	0.935	0.919, 0.950	0.0252	96.2%	90.8%	83.9%	97.9%	92.6%

## 4 Discussion

This study described the use of a training set with a relatively small sample size to construct and train an intelligent model based on Mask R-CNN that simultaneously detected the gallbladder in ultrasound images, segmented the gallbladder, and classified and diagnosed BA according to gallbladder characteristics. The performance of the model in the classification of BA in the test set with a relatively large sample size was similar to or even higher than that of pediatric radiology experts. In addition, the model still had good classification performance when the image quality was poor.

The diagnostic accuracy of radiologists on BA is highly correlated with their knowledge and clinical experience of the disease. Radiologists generally need to rely on visual senses to find the gallbladder when they diagnose BA based on the ultrasound images of the gallbladder, to determine the extent of the organ in the image, to perform the necessary measurements on the gallbladder, and to finally make a diagnosis based on the current imaging characteristics and their clinical knowledge. The above process includes three tasks: detection, segmentation of the gallbladder, and diagnosis of BA. However, actually, radiologists have a certain degree of subjectivity when interpreting the disease due to the influence of factors such as their experience, the course of the disease, and the type of pathology, and there is a lack of specific quantitative standards. Mask R-CNN has the advantage of automatic image segmentation and is one of the most pragmatic picture segmentation procedures (11). Therefore, this study introduced the Mask R-CNN model, which designs a unique three-branch structure to automatically and simultaneously complete the three tasks mentioned above after extracting the features of the image based on the CNN model.

In this article, we used the Mask R-CNN model in the ultrasonic diagnosis of biliary atresia. Compared to traditional convolutional neural network models, Mask R-CNN is more suitable for the research in this article. The main reasons are as follows: First, BA intelligent diagnostics based on ultrasound images requires two stages: object detection and diagnostic classification. When using traditional CNN methods, independent detection and diagnostic models need to be designed separately, while Mask R-CNN is a multitasking model that can simultaneously perform object detection and classification using only one model. Second, compared to traditional CNN, Mask R-CNN adds structural designs such as ROI Align and Mask branches to the model, enabling the model to intelligently diagnose ultrasound images more quickly and accurately. This model largely avoided the influence of subjective factors.

The results of this experiment showed that the accuracy of gallbladder region extraction was more than 99% independent of the model and its backbone feature extraction network, suggesting that the Mask R-CNN model has a high accuracy rate in image target extraction. Research by Wu et al. (13) shows that the accuracy of target extraction was similar using the Mask R-CNN model, independent of the backbone feature extraction network ResNet-101 or X-101-32x8d. Mariachiara Di Cosmo et al. (14) collected and marked 246 wrist ultrasound cross-sectional images and developed a Mask R-CNN model to segment the median nerve directly on the wrist ultrasound cross-sectional images. Their results showed good performance in both detection and segmentation. Our results agree with theirs.

Our results showed that model 2 was slightly better than model 1 in terms of classification accuracy in the test set, both at the image level and at the patient level, suggesting that X-101-32x8d-FPN was more appropriate to the classification of the model as the backbone feature extraction network. Our results also showed that model 2 performed slightly better than model 1 in classifying ultrasound images of different brands. Interestingly, certain brands of ultrasound images were not provided to train the model in the model training phase. However, the model still accurately classified these unfamiliar images in the model testing phase. What is more interesting is that the accuracy of model 2 was still as high as 88% when it was tested with poor-quality ultrasound images, although the diagnostic accuracy of the two models slightly decreased. This result indicated that the model constructed using Mask R-CNN still had good classification performance for poor-quality images, and the algorithm performance was relatively robust. The reasons might be as follows: ResNet-101 is a typical bottom-up deep convolutional neural network. The shallow network has a high resolution and learns the detailed features of the image. The deep network has a low resolution and learns semantic features. The higher the level, the greater the abstraction of the features, the smaller the size of the feature map, and small-sized objects are easily missed (15). The location and size of the gallbladder in this study were different, and the deep residual convolutional network was relatively easy to false alarms, making the detection results inaccurate and affecting the classification accuracy of the model. The X-101-32x8d-FPN has a characteristic pyramid structure that includes both bottom-up and top-down aspects. It still abstracts the data layer by layer from the bottom up to obtain the corresponding semantic information. The top-down process involves upsampling the abstract, semantically strengthening the high-level feature map, and then connecting the feature horizontally to the previous layer feature. The characteristic pyramid structure makes the feature map used for each layer of prediction incorporate features of different resolutions and different semantic strengths (16, 17). Thus, it is better to detect gallbladder targets of different sizes than ResNet-101 as a feature extraction and classification model (18, 19). Therefore, model 2 was slightly better than model 1 in terms of object extraction and classification.

The results of this experiment showed that the classification performance of model 2 at the image level was similar to that of the two pediatric radiology experts. However, the classification performance of model 2 at the patient level was better than that of the two pediatric radiology experts. This result revealed that the classification accuracy of the model was good. The classification accuracy is expected to play a certain auxiliary role in the diagnosis by pediatric radiologists with insufficient clinical experience in practical work. In addition, it narrowed the diagnostic gap between radiologists with different work experience and levels, which might be because the DCT was introduced in the mask representation stage. The low-precision binary gridded mask representation was replaced by a high-resolution vectorized mask representation after the DCT. The vectorized mask in the prediction process was restored to the original mask first. Then, the recovered mask was transformed from the frequency domain back to the two-dimensional image space with the two-dimensional inverse DCT (IDCT). Therefore, this method had higher accuracy than most methods (12). Zhou et al. (20) used deep learning to build an intelligent diagnosis model for BA. The sensitivity, specificity, and area under the ROC curve of BA diagnosis in the external verification set were better than the diagnostic performance of human radiologists. Nguyen et al. (21) used CNN to build an AI model to distinguish thyroid nodules as benign or malignant in 298 patients. The results showed that the overall classification accuracy of the model reached 90.88%. Yang et al. (22) used CNN to build an AI model for the auxiliary diagnosis of ultrasonic liver space-occupying lesions. The results showed that the diagnostic accuracy, sensitivity, and specificity of the model for benign and malignant liver space-occupying lesions were higher than those of a senior physician with 15 years of clinical experience. Pravat et al. (23) developed a Mask R-CNN model based on reinforcement deep learning for the real-time recognition of laryngeal cancer through the collection and annotation of a dataset of 541 laryngeal cancer images. The results showed that the diagnostic accuracy of laryngeal cancer is 98.99%. Our results agreed with those of the above studies, suggesting that the AI diagnostic model based on CNN has high diagnostic accuracy in imaging diagnosis.

The limitations of this study are the following: (1) The sample size of this study was small, and the data were far from enough. Therefore, our future plan is to collect more forward-looking data and use methods such as random rotation, flipping, and adjustment of brightness, contrast, and saturation to perform data amplification, as well as testing and verifying this model and continuously optimizing it and (2) the data included in this study were only represented by gallbladder ultrasound images, without combining them with clinical data such as liver function and stool color. Thus, our future plan is to design and build a new combination model based on the ultrasonic image features extracted by CNN and the clinical time series data features extracted by recurrent neural network to further improve the performance of the model in ultrasound diagnosis and antidiastole.

In conclusion, the BA intelligent diagnostic model based on Mask R-CNN accurately and automatically extracted the gallbladder and identified BA. Its diagnostic performance reached or was even greater than that of pediatric radiology experts. The good classification performance of model 2 suggests the potential of this non-invasive, convenient, and intelligent method to proceed and be tested in clinical trials.

## Data availability statement

The raw data supporting the conclusions of this article will be made available by the authors, without undue reservation.

## Ethics statement

The studies involving humans were approved by the ethics committee of Changsha Hospital for Maternal & Child Health Care and the ethics committee of Hunan children’s Hospital. The studies were conducted in accordance with the local legislation and institutional requirements. Written informed consent for participation was obtained from the participants’ legal guardians in accordance with the national legislation and institutional requirements.

## Author contributions

XD: Conceptualization, Data curation, Formal analysis, Funding acquisition, Investigation, Methodology, Project administration, Writing – original draft, Writing – review & editing. LY: Data curation, Methodology, Software, Validation, Writing – original draft, Writing – review & editing. WZ: Data curation, Investigation, Supervision, Writing – review & editing. HY: Data curation, Investigation, Resources, Supervision, Writing – review & editing. XX: Data curation, Investigation, Supervision, Writing – review & editing. HW: Data curation, Supervision, Writing – review & editing. WL: Data curation, Supervision, Writing – review & editing. MC: Data curation, Writing – review & editing.

## Footnotes

^1^ http://www.medcalc.org
